# What Is Causal Cognition?

**DOI:** 10.3389/fpsyg.2020.00003

**Published:** 2020-01-22

**Authors:** Andrea Bender

**Affiliations:** Department of Psychosocial Science, SFF Centre for Early Sapiens Behaviour (SapienCE), University of Bergen, Bergen, Norway

**Keywords:** causal cognition, cognitive processes, content, culture, language, knowledge accumulation, knowledge transmission

## Abstract

While gaining an understanding of cause-effect relations is the key *goal* of causal cognition, its *components* are less clearly delineated. Standard approaches in the field focus on how individuals detect, learn, and reason from statistical regularities, thereby prioritizing cognitive processes over content and context. This article calls for a broadened perspective. To gain a more comprehensive understanding of what is going on when humans engage in causal cognition—including its application to machine cognition—it is argued, we also need to take into account the content that informs the processing, the means and mechanisms of knowledge accumulation and transmission, and the cultural context in which both accumulation and transmission take place.

## Introduction

Causality is the relation between two events, one of which is the consequence (or *effect*) of the other (*cause*). Gaining an understanding of such cause-effect relations is of prime concern for humans, starting in infancy with a drive to explore one’s world and test one’s assumptions (Gopnik et al., 1999; Muentener and Bonawitz, 2017). Indeed, the ability to attain causal understanding and harness it for diagnoses, predictions, and interventions is so advantageous that it has been considered the main driving force in human evolution (Stuart-Fox, 2015; Lombard and Gärdenfors, 2017).

While understanding is arguably the key *goal* of causal cognition, its *components* are less clearly delineated. So, what exactly is causal cognition? Or rather, how should we conceptualize it from a cognitive science point of view? As will be detailed in the next section, a great deal of approaches in this field focuses on the detection of and reasoning from statistical regularities. Taking this rather narrow focus as the starting point, I will advocate a broader perspective on causal cognition, which also factors in its distinctly human characteristics, specifically the crucial roles of content, knowledge transmission, and culture. Implications for the field—including application to machine cognition—will be discussed prior to the conclusion.

## Perspectives on Causal Cognition

The preamble for this research topic outlines causal cognition as the ability “to perceive and reason about […] cause-effect relations.”^1^ This outline largely reflects what may be seen as the “standard view” in cognitive and social psychology. In the following, this view will be fleshed out, before addressing the dimensions along which it needs to be extended.

### The Standard View

Precise definitions of causal cognition are hard to come by. Scholars tend to presume that the term is self-explanatory and hence only mention in passing what they are actually focusing on. Nevertheless, a reasonably reliable impression can be gleaned from the first five publications that pop up when “causal cognition” is entered into Google Scholar (with jointly 1,280 citations in total, as of 12 August 2019, sorted by relevance).

The three publications which come from cognitive and comparative psychology cast causal cognition as the understanding of causal mechanisms (Zuberbühler, 2000; Penn and Povinelli, 2007) and as representations of the causal relation between action and outcome (Dickinson and Balleine, 2000). That is, concealed by the more generic term “causal cognition,” the subject of the respective works is actually confined to just a few aspects, each of which has an entire research tradition devoted to it: perception (Michotte, 1963; Saxe and Carey, 2006), learning (Shanks et al., 1996; Gopnik et al., 2004), and reasoning (Blaisdell et al., 2006; Waldmann, 2017).

Social psychologists add attribution, as their topic of core concern, to this range of cognitive processes (Norenzayan and Nisbett, 2000), that is, explanations of social behavior in terms of dispositional and/or contextual factors (Kelley, 1973; Choi et al., 1999). The cognitive and the social tradition essentially differ in terms of the *explanandum*—a change as the outcome of an event or of one’s actions, versus an account of why people behave in a certain way—but they both conceptualize causal cognition as consisting of mental processes.

While some scholars emphasize the domain-general nature of these processes, others consider domain boundaries to be relevant for distinguishing different types of causal cognition (Morris and Peng, 1994). And some even argue for the existence of domain-specific modules devoted to reasoning distinctly about physical, biological, and social/psychological events (Leslie, 1994; Spelke and Kinzler, 2007). Domains in this sense are defined by the distinct properties of their key entities and the causal principles accounting for their behavior. Objects in the physical domain, for instance, move when propelled by external forces in line with mechanistic principles, whereas the inhabitants of the biological domain are able to move of their own accord, in line with vitalistic principles. These different principles motivate a conceptual distinction between the constructs of *cause* (as eliciting a physical effect) versus *reason* (as motivating behavior), and between cognitive processes devoted to physical *causation* (like perception and reasoning) versus those devoted to social *agency* (like attribution and ascription of responsibility).

Only one of the five above-mentioned publications, a multidisciplinary compilation of 20 contributions on causal cognition (Sperber et al., 1995), outlines a broader range of perspectives, regarding both the processes and factors involved and the domains considered.

### A More Comprehensive View

Some core components of causal cognition, like learning based on statistical regularities, are firmly rooted in our evolutionary past: They are present in non-human animals, they are observable in human infants, and they enabled our ancestors to move out of their original habitat and spread around the globe (Bender, 2019). Even these shared roots, however, do not render causal cognition a uniform phenomenon. Relevant abilities in infants already transcend those of our closest relatives in several ways. Causal cognition in humans is characterized, *inter alia*, by the integration of content information into theory-like representations, with serious implications for processing. This role of content and the means by which it is incorporated will be outlined in more detail in the following.

#### The Role of Content for Processing

As noted above, the bulk of research on causal cognition focuses on processing while abstracting from content. As one consequence, methods prioritize artificial tasks in laboratory settings, involving toys and other stimuli designed for the very purpose of bearing no similarity to anything with which participants may be familiar (e.g., Gopnik and Sobel, 2000). Confronted with a meaningless pattern of statistical regularities, the participant’s task is to diagnose the underlying causal relations. Oddly enough, the very reason for doing so is that content plays such an overwhelming role in human causal cognition that, to be able to isolate the “pure” processes underlying it, detaching these processes from content appears indispensable.

The most abstract form of content is a structural model of the causal relations involved (e.g., whether they constitute a simple chain or a more complex network), and even rats have been shown to form such deeper causal representations, which lead their learning and reasoning (Blaisdell et al., 2006). When available, knowledge and beliefs on properties of items, on dependencies between them, or even on underlying mechanisms of causation inform these representations of structure. Pieces of knowledge are themselves embedded in mental models of how things work, which in turn guide tool use, decision-making, and problem-solving. For instance, rich knowledge on a domain affords reasoning strategies based on causal mechanisms, rather than category-based induction (Medin and Atran, 2004); and beliefs on causal mechanisms affect not only what, but also how, people decide (Kempton, 1986; Dörner, 1996; Güss and Robinson, 2014). On a higher level still, these various sorts of representations are organized by framework theories. Framework theories are ontological perspectives on the world, enriched with cultural values, that motivate interpretations, inferences, and intentions (Bang et al., 2007). They affect, for instance, how information is filed in long-term memory, whether reasoning is biased by typicality and diversity effects, or on which principles domain boundaries are drawn (Medin and Atran, 2004; ojalehto et al., 2017a,b). This need not imply that causal models are uniform or coherent; in fact, apparently incompatible accounts can co-exist in an individual’s mind and are selectively accessed depending on contextual cues (Astuti and Harris, 2008; Legare and Gelman, 2008).

In other words, content impacts on processing. If, however, the integration of knowledge and beliefs into theory-like representations is indeed so essential and decisive, accounts of human causal cognition cannot afford to disregard content.

#### The Role of Knowledge Transmission for Content

A great deal of knowledge about causation can be gleaned from an individual’s interactions with the world, and observing statistical regularities may render a reasonably accurate model of causal relations, for instance when trying to diagnose and treat a common cold. Still, accounting for the underlying mechanisms is replete with interpretation and, often enough, pure speculation. The more elaborate such accounts are, the more likely they therefore are to encompass large portions that we simply learned from other people (D’Andrade, 1995).

While learning from others is not an exclusively human ability, the extent to which our species capitalizes on it is indeed unique. Even as young children, humans pay specific attention to social cues (Kushnir et al., 2008), and when copying problem-solving behavior, they “over-imitate,” by prioritizing conventional aspects over mechanistic aspects, whether or not the former are causally relevant (Lyons et al., 2007)—a tendency that further increases into adulthood (McGuigan et al., 2011). Humans not only actively seek information, but are also willing to convey it. This willingness arises from our disposition for shared intentionality, for teaching, and for learning from teaching (Tomasello et al., 2005; Csibra and Gergely, 2009).

In contrast to the acquisition of behavioral patterns and action-based problem-solving, teaching is indispensable for the explicit transmission of knowledge, particularly for knowledge on a subject that is as invisible and ephemeral as causality (Waldmann et al., 2006). With language, humans have developed the most powerful tool in the entire animal kingdom for achieving this—a tool that young children already exploit in full when they ask for causal explanations, and persist in requesting more explanations if they are not satisfied with the previous ones (Callanan and Oakes, 1992; Frazier et al., 2009).

Given its key role for knowledge accumulation, the impact of language and its usage on causal cognition should not be underestimated. Sometimes, a linguistic label may be sufficient to serve as a cue for causal assumptions (as is the case with the common cold, which, according to popular belief, is caused by exposure to cold weather). But language use can also affect cognition more subtly, through the ways in which information about causal relations and events is encoded, or in how event descriptions are linguistically prepacked or split into their components (Wolff et al., 2009; Bohnemeyer et al., 2010). For instance, while “the climate is changing” and “humans are changing the climate” both describe the same event, the two linguistic constructions still suggest slightly diverging causal perspectives, one focusing on the event, and the other on the agent. Such modifications of the linguistic framing are able to redirect people’s attention to, in this case, event or agent (Fausey et al., 2010); to alter their inferences on causal efficacy (Kuhnmünch and Beller, 2005); to sway their memories of something they themselves observed (Loftus and Palmer, 1974; Fausey et al., 2010); or to affect their assignment of agency, responsibility, and blame (Fausey and Boroditsky, 2010; Bender and Beller, 2017).

In other words, content consists of knowledge that is socially accumulated and transmitted, frequently through explicit teaching using language. If, however, transmission is so crucial for content generation, with the means of transmission affecting causal representations and processing, accounts of human causal cognition cannot afford to disregard the role and the characteristics of the mechanisms involved.

#### The Role of Culture for Knowledge Transmission

Transmission of knowledge typically takes place within a social context. Social orientations and cultural practices therefore impact on every step of it: the bits and pieces of knowledge transmitted, the means of transmission, and the specific details of the transmission process itself.

As noted above, the bulk of people’s knowledge and beliefs is learned from others and hence bears the stamp of the cultural setting in which it emerged and is transmitted. Cultural shaping is amplified insofar as knowledge and beliefs are accumulated over time and integrated into larger models and *framework theories* (Bang et al., 2007). Cultural framework theories not only provide distinct ontological perspectives, and hence endow meaning to the causal accounts of the very same event in notably different ways, but even entail different ways of partitioning the world into domains. The ontological perspective implicit in most Western framework theories, for instance, suggests partitioning into a physical, a biological, and a social-psychological domain, largely based on properties of their key entities and on corresponding principles for agency ascription (Carey, 1996, 2009; Spelke and Kinzler, 2007). The ontological perspective implicit in Amerindian framework theories, by contrast, emphasizes interconnectedness between entities, and hence suggests principles for agency ascription that are grounded in relations rather than properties, and that give rise to domains based on communication and exchange (ojalehto et al., 2017a,b).

As a consequence, causal cognition is infused with culture. People therefore differ in whether they engage in causal considerations on a regular basis (Beer and Bender, 2015), and in how they weigh consequences versus causes (Choi et al., 2003; Maddux and Yuki, 2006). They also differ in the principles in which category and domain boundaries are grounded (ojalehto et al., 2017a,b), and in the concepts that inform their explanations (Beller et al., 2009). Even the biases that affect inferences differ across cultures (Medin and Atran, 2004; Bender and Beller, 2011). Factors contributing to these differences include, among others, the cultural shaping of the settings in which causal cognition occurs; the extent to which socialization patterns and teaching strategies encourage or discourage exploration and requests for explanation; the culture-specific organization of causally relevant knowledge, concepts, and categories; and the language-specific encoding of causal relations in grammatical structure (for reviews, see Bender et al., 2017; Bender and Beller, 2019).

In other words, knowledge transmission is ingrained in culture. If, however, the accumulation and propagation of information is so dependent on cultural practices and institutions, accounts of human causal cognition cannot afford to disregard its cultural fabric.

## Implications for Studying Causal Cognition in Humans and Machines

While causality might be objective, and our interest in it phylogenetically old, neither of the two is set in stone. As demonstrated by Iliev and colleagues (Iliev and ojalehto, 2015; Iliev and Axelrod, 2016), the extent of our concern with causality has changed over time—even over the course of just one century—and so too has the usage of the corresponding vocabulary and concepts. Here, I argue that our scientific notions of causal cognition can, and in fact must, change as well.

Research on causal cognition has typically focused on how humans gain explanations for what is going on in the world. In so doing, it often reduces causal cognition to a few cognitive processes involved in perception, learning, reasoning, and attribution, which are investigated devoid of content or context. Yet, to achieve a more comprehensive understanding of what is going on when humans engage in causal cognition, we also need to take into account the content that informs the cognitive processing, the means and mechanisms of knowledge accumulation and transmission, and the cultural context in which both accumulation and transmission take place. All of these aspects are unique to, and constitutive of, human causal cognition, and have serious implications for how we study causal cognition in humans and machines.

As a first consequence, we may wish to acknowledge more phenomena as components of causal cognition than just the inferences drawn from patterns of statistical regularities. Included should be, *inter alia*, verbal accounts, principles for categorization, tool use in daily life, problem-solving in complex situations, or judgments of blameworthiness and punishment. Concurrently, the segregation between the physical and the social domain—and hence between causation and agency—should be abolished as arguably culture-specific categorizations.

As a second consequence, we may wish to reconsider the methods we apply for investigating causal cognition. The repertoire of research strategies should be extended beyond philosophical reflections and sterile lab experiments, to also include statistical analyses of linguistic data, in-depth within-culture analyses of cognitive concepts, processes, and changes over time, ethnographic observations, or cross-cultural and cross-linguistic studies (Bender and Beller, 2016). Moreover, stronger efforts should be undertaken to increase the ecological validity afforded by our tools and settings.

A third consequence arises for attempts to model human causal cognition in machines. The recent exceptional progress in the area of artificial intelligence is largely thanks to the harnessing of deep learning for pattern recognition. Basically reflecting the “standard view” of causal cognition, this focus remains on the lowest rung of Pearl’s *Ladder of Causation* (Pearl and Mackenzie, 2018) and falls short of resembling human competences. Two of the core ingredients proposed by Lake et al. (2017) for making machines “learn and think like people” include an ability to build causal models and the grounding of learning in intuitive theories of physics and psychology (a kind of developmental “start-up software”). This emphasis on structure and content echoes insights from research on causal cognition in humans and non-human species (Pearl, 2000; Waldmann et al., 2006) and would ensure that most of the shared components of causal cognition are accounted for. Still, for modeling (uniquely) human characteristics, a further step needs to be taken: the implementation of social learning and cultural accumulation of knowledge, possibly enriched by language use (Dennett and Lambert, 2017; Tessler et al., 2017). Learning from others not only requires fewer data and occurs at a higher speed, but is also a key mechanism in diversification. As Clegg and Corriveau (2017) put it: even if the developmental “start-up software” is assumed to be universal, the “software updates” are likely shaped by culture and may over time generate distinct operating systems.

## Conclusion

To sum up, gaining an understanding of cause-effect relations is an ability in which humans clearly and strikingly outperform any other species. To a great extent, this is due to the fact that in our species, individuals are just not reliant on drawing inferences from observed statistical regularities, each on their own, but are willing and able to share their observations, inferences, and interpretations, to accumulate them over time, and to transmit them to the next generation. The content, which is so crucial in human causal cognition, is a product of culture from the very beginning, rendered possible and profoundly shaped by the fact that humans are a cultural species (Bender and Beller, 2019). While these characteristics of human causal cognition may not be considered relevant when transferring models from humans to machines—or not even desirable in some applications (Livesey et al., 2017)—it would at least be instructive to be aware of them.

## Author Contributions

The author confirms being the sole contributor of this work and has approved it for publication.

### Conflict of Interest

The author declares that the research was conducted in the absence of any commercial or financial relationships that could be construed as a potential conflict of interest.

## Footnotes

^1^https://www.frontiersin.org/research-topics/9874/causal-cognition-in-humans-and-machines
